# Changes in pre- and in-hospital management and outcomes among children with out-of-hospital cardiac arrest between 2012 and 2017 in Kanto, Japan

**DOI:** 10.1038/s41598-023-37201-1

**Published:** 2023-06-21

**Authors:** Tadashi Ishihara, Ryuji Sasaki, Yuki Enomoto, Shunsuke Amagasa, Masato Yasuda, Shima Ohnishi

**Affiliations:** 1 Department of Emergency and Critical Care Medicine, Urayasu Hospital, Juntendo University, 2-1-1, Tomioka, Urayasu, Chiba 279-0021 Japan; 2 Division of Emergency and Transport Services, National Center for Child Health and Development, Tokyo, Japan; 3 Department of Emergency and Critical Care Medicine, University of Tsukuba, Ibaragi, Japan; 4 Division of Emergency Medicine, Aichi Children’s Health and Medical Center, Aichi, Japan

**Keywords:** Epidemiology, Paediatric research, Public health

## Abstract

Previously, the SOS-KANTO 2012 studies, conducted in the Kanto area of Japan, reported a summary of outcomes in patients with out-of-hospital cardiac arrest (OHCA). This sub-analysis of the SOS-KANTO study 2017 aimed to evaluate the neurological outcomes of paediatric OHCA patients, by comparing the SOS-KANTO 2012 and 2017 studies. All OHCA patients, aged < 18 years, who were transported to the participating hospitals by EMS personnel were included in both SOS-KANTO studies (2012 and 2017). The number of survival patients with favourable neurological outcomes (paediatric cerebral performance category 1 or 2) at 1 month did not improve between 2012 and 2017. There was no significant difference in achievement of pre-hospital return of spontaneous circulation (ROSC) [odds ratio (OR): 2.00, 95% confidence interval (95% CI): 0.50–7.99, *p* = 0.50] and favourable outcome at 1 month [OR: 0.67, 95% CI: 0.11–3.99, *p* = 1] between the two studies, matched by age, witnessed arrest, bystander CPR, aetiology of OHCA, and time from call to EMS arrival. Multivariable logistic regression showed no significant difference in the achievement of pre-hospital ROSC and favourable outcomes at 1 month between the two studies.

## Introduction

Although 120,000 out-of-hospital cardiac arrests (OHCAs) occur annually in Japan, paediatric OHCAs are very rare, accounting for less than 1% of all cases^1–4^. OHCA in paediatric patients is a major global health concern, and there are approximately 1000–2000 cases in Japan annually. The survival rate is still poor, and only approximately 10–20% of paediatric patients survive 1 month after OHCA, despite a gradual improvement in the survival rate^1,3,5^. Previously, the SOS-KANTO 2002 and 2012 studies, conducted in the Kanto area of Japan, reported a summary of outcomes in OHCA patients. Compared to the SOS-KANTO 2002 study, favourable neurological outcomes at 1 month after OHCA in adult patients significantly improved in the SOS-KANTO 2012 study, which may be partly explained by the changes in Japanese law that expanded the therapeutic interventions that emergency medical service (EMS) personnel can perform in the pre-hospital setting. Under the amended laws, trained EMS personnel have been permitted to perform defibrillation since 2003, endotracheal intubation since 2004, and intravenous adrenaline administration with remote instruction from a doctor since 2006 in adult OHCA patients but not in paediatric OHCA patients aged < 8 years. Therapeutic procedures by EMS for paediatric OHCA patients aged < 8 years did not change between the study periods. In fact, EMS personnel can only perform bag-valve-mask ventilation and chest compression on paediatric OHCA patients during transport to the hospital. In some areas, ambulances with doctors or helicopters with doctors are in operation; doctors can perform endotracheal intubation or adrenalin administration via the intravenous or intraosseous route in the pre-hospital setting^5–8^.

This sub-analysis of the SOS-KANTO study 2017 aimed to evaluate the changes in pre-hospital procedures performed by EMS personnel or physicians and in-hospital treatment, and the outcomes of paediatric OHCA patients, by comparing the SOS-KANTO studies from 2012 and 2017.

## Results

**Figure 1:**
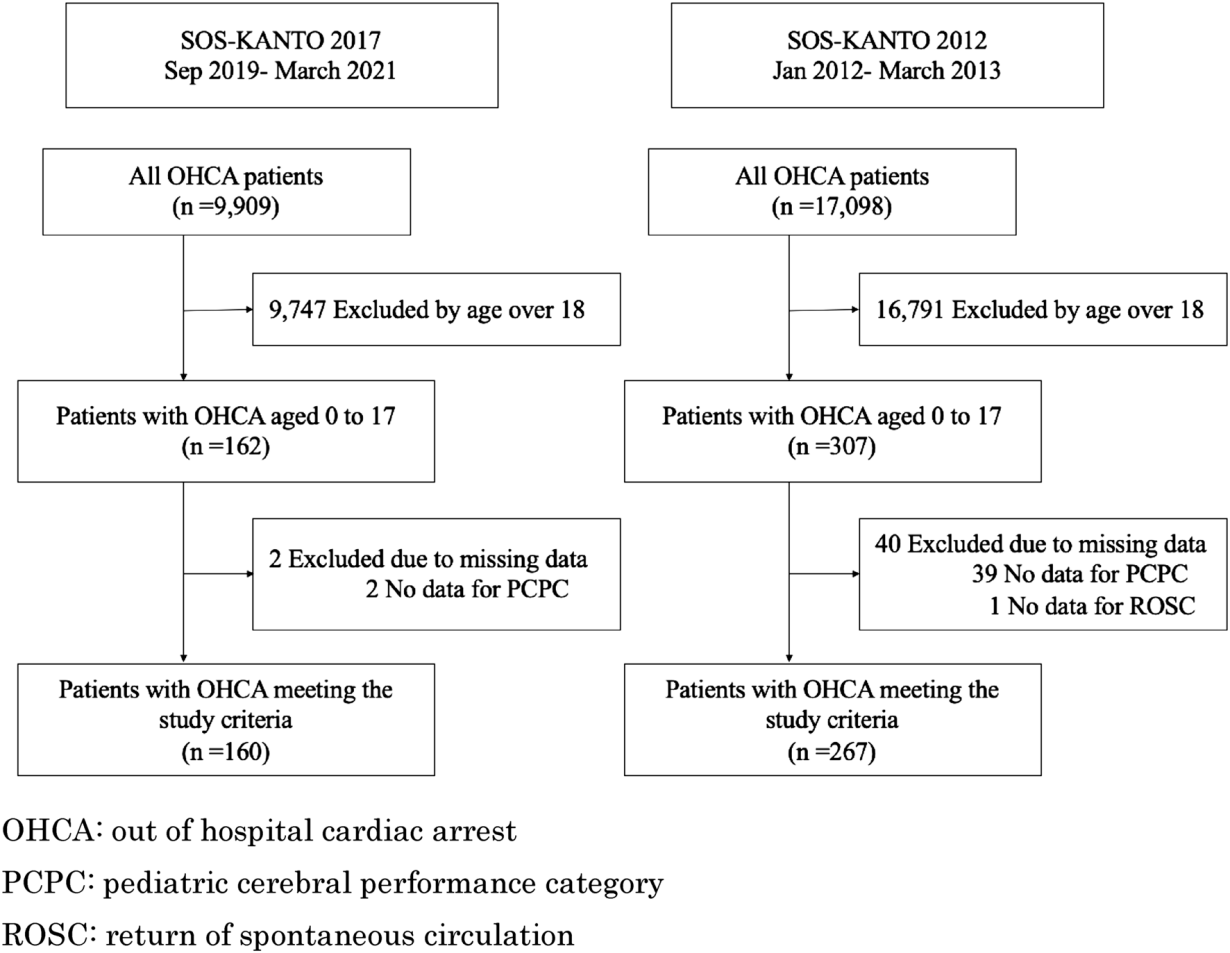
Study patients. *OHCA* out of hospital cardiac arrest, *PCPC* pediatric cerebral performance category, *ROSC* return of spontaneous circulation.

A total of 267 of the 17,098 OHCA patients from the SOS-KANTO 2012 study and 160 of the 9909 OHCA patients from the SOS-KANTO 2017 study met the inclusion criteria (Fig. 1).

Table 1 summarizes the baseline patient demographics and characteristics of the SOS-KANTO 2012 and 2017 groups. The median age was significant higher in the SOS-KANTO 2017 study than that in the SOS-KANTO 2012 study (3.5 vs. 1, *p* < 0.05). There were significant differences in arrest location and aetiology of OHCA between the two groups. (*p* < 0.05). Most paediatric patients [115 (71.9%) from the SOS-KANTO 2017 study and 165 (58.1%) from the SOS-KANTO 2012 study] suffered OHCA at home, and some experienced OHCA in a public place.

**Table 1:** Patient’s demographics, event and characteristics in SOS-KANTO 2012 and 2017.

	2017 (n = 160)	2012 (n = 267)	p-values
Age (years), median (IQR)	3.5 (0–15)	1 (0–11)	< 0.05*
Gender (male, %)	91 (56.9)	155 (58.1)	0.84
Arrest location			< 0.05*
Home (%)	115 (71.9)	165 (58.1)	
School (%)	5 (3.1)	26 (9.7)	
Public (inside) (%)	12 (7.5)	41 (15.4)	
Public (outside) (%)	12 (7.5)	11 (4.1)	
Others (%)	16 (10)	4 (1.5)	
Witnessed by layperson (%)	55 (34.4)	113 (42.3)	0.13
Bystander CPR performed (%)	79 (49.4)	115 (43.1)	0.23
Bystander CPR by healthcare personnel (%)	12 (15.2)	11 (9.6)	0.26
Public access AED (%)	6 (3.7)	14 (5.2)	0.64
Shocked by AED (%)	4 (2.5)	7 (2.6)	1
Initial documented rhythm			0.75
Shockable (%)	3 (1.9)	7 (2.6)	
Non-shockable (%)	157 (98.1)	260 (97.4)	
Etiology of OHCA			< 0.05*
Cardiac cause (%)	55 (34.4)	57 (21.3)	
Non-cardiac, intrinsic cause (%)	21 (13.1)	88 (33.0)	
Extrinsic cause (%)	70 (43.8)	108 (40.4)	
SIDS (%)	14 (8.8)	14 (5.2)	

*IQR* interquartile range, *CPR* cardiopulmonary resuscitation, *AED* automated external defibrillator, *OHCA* out of hospital cardiopulmonary arrest, *SIDS* sudden infant death syndrome.

*p < 0.05.

Table 2 shows the results of pre-hospital interventions. Bag-valve mask ventilation was the most frequently used method for advanced airway management by EMS personnel [131 (81.9%) in SOS-KANTO 2017 and 227 (85.0%) in SOS-KANTO 2012] followed by supraglottic airway device. Only 1 (0.6%) and 6 (2.2%) patients underwent endotracheal intubation in SOS-KANTO 2017 and 2012, respectively, but the difference was not significant. There were significant differences between the groups in the number of adrenalin doses, release of asphyxia, time from call to EMS arrival, and presence of physician at the pre-hospital scene.

**Table 2:** Pre-hospital interventions in SOS-KANTO 2012 and 2017.

	2017 (n = 160)	2012 (n = 267)	p-values
Intervention at pre-hospital			
Advanced airway management			0.30
Bag-valve mask (%)	131 (81.9)	227 (85.0)	
Supraglottic airway device (%)	24 (15.0)	31 (11.6)	
Intubation (%)	1 (0.6)	6 (2.2)	
Others (%)	4 (2.5)	3 (1.1)	
Intravenous line (%)	13 (8.1)	10 (3.7)	0.08
Adrenalin administration (%)	13 (8.1)	10 (3.7)	0.08
Time from call to adrenalin administration (min, IQR)	33 (26–42)	34 (28–41)	0.48
Number of adrenalin doses (median, IQR)	3 (1–3)	0 (0–2)	< 0.05*
Automated chest compression (%)	3 (1.9)	5 (1.9)	1
Defibrillation (%)	7 (4.9)	13 (4.9)	1
Release of asphyxia	6 (3.8)	25 (9.4)	< 0.05*
Time from call to EMS arrival (min, IQR)	8 (6–10)	7 (6–9)	< 0.05*
Physician present at pre-hospital (%)	21 (13.1)	12 (4.5)	< 0.05*

*EMS* emergency medical service, *IQR* interquartile range.

*p < 0.05.

Table 3 summarizes the in-hospital interventions. Fifteen (9.4%) and 11 (4.1%) patients achieved ROSC before reaching the hospital, and 25 (15.6%) and 51 (19.1%) patients achieved ROSC after reaching the hospital in SOS-KANTO 2017 and 2012, respectively. There was no significant difference in ROSC rate between the groups. There were also no significant differences in in-hospital interventions, such as endotracheal intubation, defibrillation, adrenalin administration, or target temperature management.

**Table 3:** In-hospital interventions in SOS-KANTO 2012 and 2017.

	2017 (n = 160)	2012 (n = 267)	p-values
ROSC information			0.08
Pre-hospital ROSC (%)	15 (9.4)	11 (4.1)	
In-hospital ROSC (%)	25 (15.6)	51 (19.1)	
No chance of ROSC (%)	120 (75.0)	206 (76.8)	
In-hospital Intervention			
Intubation			0.53
In-hospital	124 (77.5)	202 (75.7)	
Pre-hospital by EMS	1 (0.6)	6 (2.2)	
No-intubation	35 (21.9)	59 (22.1)	
Automated chest compression (%)	5 (3.1)	3 (1.1)	0.15
Defibrillation (%)	7 (4.4)	12 (4.5)	0.97
Adrenalin administration (%)	126 (78.8)	215 (80.5)	0.71
Number of adrenalin doses (median, IQR)	5 (3–8)	5 (3–8)	0.45
Amiodarone (%)	2 (1.2)	2 (0.7)	0.63
Lidocaine (%)	1 (0.6)	2 (0.7)	1
Atropine (%)	1 (0.6)	16 (6.0)	< 0.05*
Vasopressin (%)	0	2 (0.7)	0.53
TTM ≤ 34 ℃ (%)	6 (3.8)	18 (6.7)	0.28

*ROSC* return of spontaneous circulation, *EMS* emergency medical service, *IQR* interquartile range, *TTM* target temperature management.

*p < 0.05.

Table 4 shows the outcomes in the SOS-KANTO 2017 and 2012 studies. The number of surviving patients with favourable neurological outcome (PCPC 1 or 2) at 1 month did not improve between 2012 and 2017. In addition, the survival rate at 1 month showed a slight but not significant increase. The most frequent outcome in both studies was PCPC 6 (brain death or death).

**Table 4:** Outcome in SOS-KANTO 2012 and 2017.

	2017 (n = 160)	2012 (n = 267)	p-values
One month survival	19 (11.9)	25 (9.4)	0.42
Favorable neurological outcome (PCPC1 or 2, %)	4 (2.5)	7 (2.6)	1
PCPC scale category after 1 month			0.39
PCPC 1 (%)	4 (2.5)	6 (2.2)	
PCPC 2 (%)	0	1 (0.4)	
PCPC 3 (%)	1 (0.6)	0	
PCPC 4 (%)	5 (3.1)	11 (4.1)	
PCPC 5 (%)	9 (5.6)	7 (2.6)	
PCPC 6 (%)	141 (88.1)	242 (90.6)	

*PCPC* pediatric cerebral performance category.

*p < 0.05.

The results of the multivariable logistic analysis for the achievement of pre-hospital ROSC and favourable neurological outcome are shown in Table 5. There was no significant difference in achievement of pre-hospital ROSC (OR: 2.00, 95% CI: 0.50–7.99, *p* = 0.50) and favourable outcome at 1 month (OR: 0.67, 95% CI: 0.11–3.99, *p* = 1) between the two study periods, matched by age, witnessed arrest, bystander CPR, aetiology of OHCA, and time from call to EMS arrival.

**Table 5:** Logistic regression analysis.

	OR	95% CI	p-value
Achieve of pre-hospital ROSC (unmatched)	2.62	1.05 to 6.57	< 0.01*
Achieve of pre-hospital ROSC (matched)	2.00	0.50 to 7.99	0.50
Favorable neurological outcome at 1 month (unmatched)	0.95	0.27 to 3.31	< 0.01*
Favorable neurological outcome at 1 month (matched)	0.67	0.11 to 3.99	1

*OR* odds ratio, *CI* confidence interval, *ROSC* return of spontaneous circulation.

*P < 0.01.

## Discussion

This study compared epidemiological data on cardiopulmonary resuscitation in the Kanto area between 2012 and 2017. To our knowledge, this is the first large study to compare the outcome of paediatric OHCA in different periods in the Kanto area. Although there are several reports on paediatric OHCA based on analyses of the National Utstein Registry, a nationwide population-based OHCA registry, they all report neurological outcomes by cerebral performance category (CPC), and not PCPC^4,5,7^. The number of OHCA patients between SOS-KANTO 2012 and 2017 studies differ significantly. It is assumed that the number of OHCA patients were decreased and registration was delayed due to pandemic of COVID-19. In fact, although the study period was longer than previous study, the number of patients enrolled in the registry was half of previous study. According to the demographic data in Japan, the number of infant deaths has been decreased from 2292 in 2012 to 1512 in 2020, and this may be one of the factors. An analysis of SOS-KANTO registry data between 2012 and 2017 revealed no significant differences in one-month survival, with favourable neurological outcome set as the primary outcome, during the last 5 years of the period. Despite analysing the data by multivariable logistic regression and matching the groups by age, witnessed arrest, bystander CPR, time from call to EMS arrival, and aetiology of OHCA, there were no significant differences in favourable neurological outcome or achievement of pre-hospital ROSC between the two periods.

Although there were no significant differences in bystander CPR or witnessed arrest, which have been suggested as a predictors for favourable neurological outcome after OHCA, between the two periods, the rate of bystander CPR showed a slight increase from 43.1% in 2012 to 49.4% in 2017^9–11^. There was no significant difference in one-month survival, with favourable neurological outcome set as the primary outcome, between two periods. In fact, there were no significant differences in the procedures performed at the pre-hospital scene by EMS personnel between the two periods. Pre-hospital care may be related to the lack of improvement in survival outcome. A comparison of SOS-KANTO 20202 and 2012 study of adults, the survival rate with favorable neurological outcome has been improved due to the approval of tracheal intubation in 2004, the administration of adrenaline in 2006 for EMS personnel, and widespread use of AED^9,10^. Since there was no change in pre-hospital care provided by EMS personnel for children between two study periods, it could be assumed that there was no change in outcomes. The rate of physician presence at the pre-hospital scene was higher in 2017 than that in 2012 (13.1% vs 4.5%, *p* < 0.05). The success rate of ROSC at the pre-hospital scene was higher in 2017 than in 2012. Although physician presence at the pre-hospital scene may have affected the success rate of ROSC, no significant differences were observed in the rate of advanced airway management or time to adrenaline administration, which limits the generalization of these findings. The success rate of ROSC at the pre-hospital scene was higher in 2017, but it was not linked to favourable neurological outcome.

Although limited to adults with OHCA, a comparison of the SOS-KANTO 2002 and 2012 studies showed improved favourable neurological outcome in 2012 compared to that in 2002^10^. Increasing the frequency of bystander CPR and automated external defibrillator (AED) use and improving the therapeutic management after ROSC would be particularly beneficial to patients^9,10^. In this study period, there was no significant increase in the rate of bystander CPR or in the use of AED, even though CPR and AED are thought to have been become more popular among the general population^9^.

A predictor for the success of resuscitation at the pre-hospital scene in paediatric OHCA has not been yet established. Age, bystander CPR, and earlier initiation of CPR by EMS personnel are potential predictors for the success of resuscitation at the pre-hospital scene^12–21^. Although there are several reports of shockable rhythm as initial rhythm, related to 1-month survival or favourable neurological outcome, the reported rate of shockable rhythm in paediatric OHCA patients in Japan is very low (3.9–4.9%)^14,16,19^. In this study, the rate of paediatric OHCA patients with shockable rhythm was also very low (1.9% in 2017 and 2.6% in 2012). This may explain the low rate of favourable neurological outcome in the SOS-KANTO study in both periods.

Some previous studies on paediatric OHCA based on the National Utstein Registry reported no significant difference in favourable neurological outcome in paediatric OHCA patients between advanced airway management (AAM), such as endotracheal intubation (ETI) or supraglottic airway device (SGA), and bag-valve-mask ventilation (BVM) management at the pre-hospital scene^5,7,8,22,23^. In this study, there was also no significant difference between the two periods in AAM at the pre-hospital scene. A previous systematic review revealed no significant difference in survival discharge or favourable neurological outcome between AAM and BVM^24^, which suggests the importance of implementing reliable BVM ventilation rather than unreasonable AAM.

Pre-hospital AAM has an important role in paediatric OHCA since the respiratory aetiology is a major cause of this condition^15,25–27^. A previous report indicated that the neurological outcome of paediatric OHCA may be improved by ensuring reliable airway management by AAM in cases of non-cardiac aetiology^8^. For ensuring the airway management, EMS personnel can use ETI or SGA. Although ETI is more frequently used than SGA for airway management in the US, SGA is used more frequently than ETI in Japan^23,28^. It is because that the EMS system in Japan is different from that in other countries^6^. There are age restrictions on the procedures that can be performed by EMS personnel at the pre-hospital scene. For instance, EMS personnel is not allowed to perform ETI in patients younger than 8 years old by Japanese law.

No RCTs have been conducted on the effect of adrenaline administration in paediatric OHCA patients, and there are only a few observational studies on this subject^29,30^. In this paediatric OHCA study, the adrenaline effect was limited due to the small sample size and the fact that only descriptive evidence was provided. Instead, the current recommendation of adrenaline administration for paediatric cardiac arrest is based on adult OHCA studies. Therefore, we considered the evidence of adult OHCA for paediatric OHCA. Although, there is substantial evidence about the effect of adrenaline on adult cardiac arrest, the prognosis and neurological status after a long-term period remains controversial^31^. Although more doses of adrenaline were administered in 2017 than in 2012, there were no significant differences between the two periods in the time elapsed from call to adrenaline administration, time from call to EMS arrival, intervention at hospital, 1-month survival rate, or favourable neurological outcome. In addition, our results revealed a much lower proportion of adrenaline administration in the pre-hospital setting than the proportion reported in other countries. A previous study reported a proportion of 8.3% of adrenaline administration from 2007 to 2010^6^. In our study, the proportion rate of adrenaline administration was also low (8.1%), and it has not changed in nearly decade. On the other hand, the proportion rate of adrenaline administration in the US is higher than that of our study (73.3% vs 8.1%)^32^. The difference might be due to differences in the EMS system, including the fact that there are age restrictions for intravenous access placement and adrenaline administration performed by EMS personnel in Japan. EMS personnel are not allowed to perform intravenous or interosseous access placement in paediatric patients aged < 8 years. Currently, EMS personnel can only provide BVM ventilation and chest compression to paediatric OHCA patients. The procedures that EMS personnel can perform have not changed since the last study period, which might explain the lack of significant differences between the two periods in terms of favourable neurological outcome or achievement of pre-hospital ROSC in paediatric OHCA patients. One of the special notes in this study is that one month survival rate was lower than other OHCA studies. One month survival rate was 11.9% in our study, but one month survival rate in therapeutic Hypothermia after Pediatric Cardiac Arrest Out-of-Hospital (THAPCA-OH) trial is significant differ at 33.4%^33^. The THAPCA clinical trial is a high-quality RCT that provides well-informed, evidence based information and is well worth reporting. Although a direct comparison between SOS-KANTO 2017 study and THAPCA-OH trial is not possible, there are some factors that could account for difference in survival rate. It has been reported that a shorter time to adrenaline administration improves the ROSC rate in pediatric OHCA, but in Japan, pre-hospital care of EMS personnel is legally restricted, EMS personnel cannot administer adrenaline, and must transport the patients with only BVM ventilation and chest compression^32,34^. The median time of first adrenaline administration in our study was 33 min, which differs significantly from previous study, median time was 7.6 min^34^. The rate of ROSC and rate of survival to hospital discharge are very low, because EMS personnel transport all OHCA patients to the hospital, unless a cadaveric reaction such as rigor mortis or cadaveric spots is manifested, regardless of the patient age in Japan^12–14,35^. It is assumed that these factors influence the difference in survival rate.

Our study has some strengths. First, our study used PCPC for assessing neurological outcome, which is different from the methods used in previous studies analysing the National Utstein Registry that reported neurological outcomes in paediatric OHCA patients using CPC. As the National Utstein Registry does not collect PCPC data, the SOS-KANTO study data are more suitable for assessing neurological outcomes in paediatric OHCA patients, and hence in this study, we used PCPC for neurological assessment. Another strength of our study was the comparison between paediatric patients from two large databases from 2012 and 2017 that were prospectively collected by EMS personnel and hospital staff in the same area.

There are also some limitations to our study. First, the SOS-KANTO study did not include all hospitals in the Kanto area; therefore, there may be a selection bias towards more academically focused or resource-rich hospitals that were able to join the SOS-KANTO study. Second, the procedures that EMS personnel are allowed to perform are slightly different from other countries; particularly, the EMS personnel are not permitted to perform interosseous access for OHCA patients, regardless of adults or children^8,36^. There is confounding bias, because the EMS personnel are not permitted to perform intravenous placement or adrenaline administration in paediatric OHCA patients aged < 8 years. Third, the SOS-KANTO study used retrospective data, and the accuracy, precision, or failure of the procedures performed by EMS personnel in the pre-hospital setting are not completely clear. Fourth, the frequency of autopsy in Japan is very low, and the aetiology of paediatric OHCA is sometimes diagnosed tentatively. Finally, as with all epidemiological studies, the integrity, validity, and ascertainment bias of the data are potential limitations.

In conclusion, this is the first large study to compare the outcome of paediatric OHCA in different periods in the Kanto area by PCPC. There was no significant difference in the achievement of pre-hospital ROSC and favourable outcomes at 1 month between the two study periods analysed by multivariable logistic regression. It is revealed that there was no change in outcomes between two study periods, and that outcomes differed significantly from the rest of the other countries. Besides, there was no change in pre-hospital care over this time period. Hopefully, our study and further research provide the paradigm shift in pre-hospital care by EMS personnel.

## Methods

### Study design

The SOS-KANTO 2012 study was undertaken in the Kanto area of Japan and included 68 hospitals. It was supported by the Kanto Regional Group of the Japanese Association for Acute Care Medicine between January 2012 and March 2013^9^. More recently, the SOS-KANTO 2017 study was a prospective survey that aimed to collect the data on patients with OHCA in the Kanto area, between April 2019 and July 2021, with the participation of 46 hospitals. The study period of SOS-KANTO 2017 was longer than that of SOS-KANTO 2012 as it was not possible to collect enough data during the coronavirus disease pandemic. This study was approved by the relevant institutional review boards of all 46 hospitals.

### Definitions and data collection

Cardiac arrest was defined as the absence of cardiac activity with pulse and normal breathing^37^. All OHCA patients who were transported to the participating hospitals by EMS personnel were included in both SOS-KANTO studies (2012 and 2017). This sub-analysis of the SOS-KANTO 2017 study included only paediatric patients (aged < 18). Pre- and in-hospital treatment were provided by EMS personnel, physicians and other healthcare providers^38,39^. EMS personnel collected the pre-hospital information and Utstein style outcome reports^37^. They collected information on the patients’ characteristics, initial cardiac rhythm, and the time course of resuscitation. Additional information included whether the arrest was witnessed by a bystander, whether a bystander initiated cardiopulmonary resuscitation (CPR), whether the patient was intubated, whether epinephrine was administered, and whether the patients achieved return of spontaneous circulation (ROSC) before arriving at the hospital.

The causes of cardiac arrest were defined by the physician at the hospital and were diagnosed as cardiac aetiology unless an obvious non-cardiac aetiology (i.e., cerebrovascular disease, respiratory disease, severe trauma, drowning, asphyxiation, or drug overdose) was observed.

A physician or institutional researcher collected the following in-hospital information; medications for resuscitation, interventions, laboratory data, length of hospital stay, and neurological outcome at 1 month from the cardiac arrest. Neurological outcome was evaluated using the Paediatric Cerebral Performance Category (PCPC) scale (1: good cerebral performance, 2: mild cerebral disability, 3: moderate cerebral disability, 4: severe cerebral disability, 5: come/vegetate state, 6: brain death/death)^37^.

### Ethical approval and consent to participate

The Ethics Committee of Juntendo University Urayasu Hospital approved the SOS-KANTO 2017 study data analysis (approval number: 1-022) The requirement for patient or parent consent was waived by the Ethics Committee of Juntendo University Urayasu Hospital as this was an epidemiologic study that used anonymized data. We used the STROBE statement as a guide for reporting this study.

This study was approved by the Institutional Review of Juntendo University Urayasu Hospital, Chiba, Japan (1-022) and was conducted in accordance with the principles outlined in the 1964 Declaration of Helsinki and its later amendments.

### Selection of participants

We evaluated paediatric patients aged < 18 years with cardiac arrest, who received CPR by the EMS personnel and were transported to the participating hospitals. We excluded cases with missing data on the following variables: (1) main outcomes at 1 month survival with favourable neurological outcome or ROSC; (2) time course of CPR by EMS personnel or physicians; (3) arrest witness or bystander CPR initiation; and (4) in-hospital treatment.

### Outcome measures

The primary outcome measure was paediatric patient survival with favourable neurological outcome at 1 month from cardiac arrest, which was defined as PCPC Scale 1 or 2. The secondary outcome was the success rate of ROSC.

### Statistical analysis

To display patient data, median with interquartile range (IQR) was used for numerical variables. Baseline patient characteristics from the SOS-KANTO 2012 and 2017 studies were compared using the chi-square test or Fisher’s exact test for frequencies, and a* t*-test or Mann–Whitney *U*-test was used for continuous variables, as appropriate. Differences were considered significant when the *P*-value was less than 0.05. To assess the independent effect of the study, multivariable logistic regression analysis was performed on the success rate of ROSC and favourable neurological outcome at 1 month. Age, witness arrest, bystander CPR, aetiology of OHCA, and time from call to EMS arrival were included as variables in the analysis. Data management and statistical analysis were performed using the EZR software (Y Kaneda, Saitama Medical Centre, Jichi Medical University, Saitama Japan).

## Data Availability

The datasets generated and/or analysed during the current study are not publicly available due to including of privacy but are available from the corresponding author on reasonable request.

## Abbreviations

OHCA: Out-of-hospital cardiac arrest
EMS: Emergency medical service
CPR: Cardiopulmonary resuscitation
ROSC: Return of spontaneous circulation
PCPC: Paediatric cerebral performance category
IQR: Interquartile range
CPC: Cerebral performance category
AED: Automated external defibrillator
AAM: Advanced airway management
ETI: Endotracheal intubation
SGA: Supraglottic airway device
BVM: Bag-valve-mask
